# Reduction of peripersonal comfort space correlate with eating disorder symptoms in young adolescents: a network analysis approach

**DOI:** 10.3389/fpsyg.2024.1420247

**Published:** 2024-09-05

**Authors:** Beatriz Pereira Da Silva, Andrea Escelsior, Monica Biggio, Alessio Zizzi, Martino Belvederi Murri, Riccardo Guglielmo, Alberto Inuggi, Federico Delfante, Giacomo Marenco, Mario Amore, Gianluca Serafini

**Affiliations:** ^1^Department of Neuroscience, Rehabilitation, Ophthalmology, Genetics, and Mother-Child, School of Medical and Pharmaceutical Sciences, University of Genoa, Genova, Italy; ^2^San Martino Hospital (IRCCS), Genova, Italy; ^3^Department of Neuroscience and Rehabilitation, University of Ferrara, Ferrara, Italy

**Keywords:** peripersonal space, network analysis, adolescents, psychopathology, eating disorders

## Abstract

**Background:**

Peripersonal Space (PS) is represented as the immediate area surrounding an individual. The extent of PS changes in relation to several factors, including emotional states, type of relationship or psychopathology. Attachment anxiety has an impact on the social adaptability of peripersonal space and anxiety and fear are associated with an expansion of peripersonal space, possibly serving as a mechanism of self-protection. Peripersonal space appears to be intricately linked to various psychiatric conditions like anxiety disorders and converging evidence suggests that social maladjustment may predict or exacerbate eating disorder symptoms expression.

**Methods:**

Fifty-eight healthy adolescents (38F, 20M) performed a comfort distance estimation task to assess peripersonal space. The Adolescent/Adult Sensory Profile (AASP) was used to assess sensory profiles and the SAFA protocol to investigate psychopathological aspects. Data was analysed using Network Analysis, estimating a Gaussian Graphical Models with a Bayesian approach.

**Results:**

We found that the task related to comfort estimation distance demonstrated a correlation with the visual scale of the Adolescent/Adult Sensory Profile (AASP). Additionally, a correlation was observed with the Eating Disorder scale of the SAFA protocol. The touch scale also was negatively correlated with Eating disorder symptoms but not with the comfort estimation task.

**Conclusion:**

Our results demonstrate a relation between peripersonal space and eating disorder symptoms in healthy adolescents in line with previous findings in adults with eating disorders diagnosis. These findings suggest that socio-emotional difficulties may be possible precursors or reinforce for the development of an eating disorder symptoms.

## 1 Introduction

Peripersonal Space (PPS) refers to the spatial region that exists between the body and objects in close proximity (Rizzolatti et al., 1997). PPS can be represented as the immediate area surrounding an individual, typically within arm’s reach or within the distance at which objects can be grasped (Brozzoli et al., 2011; Biggio et al., 2019). It has been argued that the incorporation of external stimuli within the PPS significantly contributes to the development of body self-consciousness, involving a clear demarcation between oneself and others (Ardizzi and Ferri, 2018). Furthermore, PPS is not solely confined to sensorimotor integration with the environment; it has been indicated as playing a central role in the development of higher-level cognitive functions. As suggested by Teneggi et al., the dimensions of PPS are intricately shaped by social cognition and interpersonal relationships (Teneggi et al., 2013).

PPS appears to be intricately linked to various psychiatric conditions. In a study using an augmented reality version of a multisensory interaction task, von Mohr et al., discovered that attachment anxiety has an impact on the social adaptability of PPS (von Mohr et al., 2023). Individuals showing symptoms frequently linked with trauma-related disorders like PTSD show early alterations of PPS (Bogovic et al., 2013). The observed connections between PPS and psychiatric conditions suggest a potential clinical significance of PPS in the pathophysiology of mental disorders (Longo et al., 2024).

In particular, anxiety and fear are associated with an expansion of PPS boundaries, possibly indicating a mechanism of self-protection (Biggio et al., 2023). Social anxiety particularly affects young people, who might also show significant alterations in the development of PPS. Attachment anxiety may be associated with altered PPS, reflecting ongoing adaptations in processes related to social acceptability (Spaccasassi and Maravita, 2019). Understanding the impact of anxiety on PPS might provide useful insights to inform therapeutic approaches for social anxiety or subclinical social difficulties during the developmental age (e.g., individuals experiencing difficulties in social interactions not yet fulfilling criteria for social anxiety disorders). Additionally, the alterations in PPS associated with trauma-related disorders open avenues for further exploration into the role of PPS in the manifestation and treatment of psychiatric symptoms.

We hypothesize that the extent and characteristics of PPS in healthy adolescents may be associated with the risk of developing future psychopathology in adulthood. The primary hypothesis of our study posits that alterations in PPS may already be present in healthy adolescents who are at risk of developing psychopathology in adulthood, particularly those grappling with anxiety.

To test our hypothesis, we tested the extension of comfort PPS in a group of healthy adolescents with a comfort distance estimation task. Subsequently, we have correlated these results with the Adolescent/Adult Sensory Profile (AASP) senses subscale and the Child and Adolescent Psychiatric Self-Administrated Scales (SAFA). This research aims to explore potential early indicators of psychopathology in adolescents in PPS alterations. The findings may contribute to the development of targeted interventions and preventive measures, ultimately supporting the mental well-being of young individuals.

## 2 Methods

### 2.1 Participants and measures

Fifty-eight adolescents (38 females, 20 males) aged between 12 and 13 years were recruited from three classes of a secondary school in Italy. Our study aimed to include a comprehensive sample of students from a particular age group within a school to ensure uniformity and minimize confounding variables related to age. By selecting all students of the same age, we intended to create a homogenous group for our analysis.

The primary criterion for participation was the ability to use a personal computer, reflecting a practical competency relevant to our research objectives. This criterion was selected to ensure that all participants could engage effectively with the digital tools required for the study. There were no other *a priori* criteria for inclusion, allowing for a diverse and representative sample of students within the specified age group.

First, the extension of participants’ PPS was tested with a standardized procedure. Then, participants underwent the assessments with the Adolescent/Adult Sensory Profile (AASP) to evaluate sensory profiles, then they were administered the SAFA protocol to investigate psychopathology. The study adhered to ethical standards, as evidenced by the approval from the Ethical Committee and the provision of written consent by the parents of the participating adolescents. The welfare and privacy of the participants were prioritized throughout the research process.

#### 2.1.1 Peripersonal space evaluation task

The Peripersonal space evaluation task consisted in watching videos showing two avatars walking towards each other. The participant was asked to identify themself with the indicated avatar, then the other avatar approached the participant’s avatar. Subjects were asked to press the spacebar to stop the video when the moving avatar reached the target distance, namely the distance beyond which the position of the other avatar would create discomfort to the participant, following these instructions: “stop the movement as soon as the distance between you and the other avatar makes yourself feel uncomfortable” (Biggio et al., 2022).

#### 2.1.2 Sensory profiles evaluation

The Adolescent/Adult Sensory Profile (AASP) is a self-report questionnaire, comprising 60 items with responses on a 5 points Likert scale (from 1 = “Almost Never” to 5 “Almost Always”). The AASP is used to characterize the sensory profiles. Participants answer questions about sensory experiences in daily life with regard to the different sensory systems (Engel-Yeger et al., 2016). For this study we computed the sensory modality subscale scores, i.e., Taste, Kinaesthesia, Visual, Auditory, Smell, Touch. Higher scores on a modality or sense represent increased sensitivity or responsiveness within that sensory system, indicating that the individual may have a heightened or more pronounced reaction to sensory stimuli.

#### 2.1.3 Psychopathology evaluation

The Child and Adolescent Psychiatric Self-Administrated Scales (SAFA) is a coordinated battery of rating instruments to explore a broad range of internalizing psychopathology symptoms. The SAFA has been validated according to the criteria of DSM-5 (Cianchetti, 2001) and investigates: (1) SAFA-A: anxiety symptoms (Generalized anxiety, Social anxiety, Separation anxiety, School related anxiety); (2) SAFA-D: depressive symptoms (Depressed mood, Anhedonia, Irritable mood, Feeling of inadequacy, Insecurity, Guilt, Desperation); (3) SAFA-O obsessive-compulsive symptoms (Obsessive thoughts, Compulsions, Rupophobia, Order, Doubt); (4) SAFA-P eating disorders (Bulimic behaviors, Anorexic behaviors, Body acceptance, Psychological aspects); (5) SAFA-S somatic and hypochondriac symptoms (Somatic symptoms, Hypochondria). The SAFA items use a 3 points scale (1 = “True,” 2 = “Neutral,” 3 = False) except on the SAFA-S scale which uses a frequency scale (1 = “Often,” 2 = “Sometimes,” 3 = “Never”). The table below contains a summary of the SAFA protocol, the total scores are obtained by adding the scores of the subscales (Table 1).

**Table 1:** SAFA’s Items number and subscales.

SAFA scale	Number of items	Subscales	Item example
SAFA-A	50	Generalized anxiety Social anxiety Separation anxiety School related anxiety	I feel quite calm and relaxed I do not like playing with others I feel bad when my parents are not with me I often feel very nervous when I have to go to school
SAFA-D	56	Depressed mood Anhedonia Irritable mood Feeling of inadequacy Insecurity Guilt Desperation	I am usually happy and cheerful I am not interested in doing anything, not even playing If someone bothers me, I get angry immediately I am happy with myself I often do not feel confident about myself I often deserve to be punished I think life is beautiful
SAFA-O	38	Obsessive thoughts Compulsions Rupophobia Order Doubt	I am tormented by the idea that something bad might happen I often feel the need to repeat certain actions or thoughts to prevent something bad from happening I always feel like my hands are dirty It bothers me a lot if my things are not exactly in their place I often doubt whether I have done things right
SAFA-P	30	Bulimic behaviors Anorexic behaviors Body acceptance Psychological aspects	I often eat excessively I often avoid eating I am not satisfied with my physical appearance I am not as good as I should be
SAFA-S	25	Somatic symptoms Hypochondria	I always feel tired from the moment I wake up in the morning I am very afraid of illnesses

## 3 Data analyses

We explored the association between psychopathological aspects and sensory profile based on the Network Theory of Mental Disorders (Borsboom, 2017). In network analyses, symptoms (variables) are represented as nodes, and the causal relationships between them are represented as edges (lines connecting them). Thicker edges indicate stronger associations; those can be positive (green) or negative (red). Psychopathology Network Analyses are based on the Gaussian Graphical Model (GGM), which identifies significant conditional dependencies between nodes corresponding to rating scale items or subscales. The pairwise relations are akin to partial correlations, controlling for the effects of all other variables in the model (Williams, 2021). Age and sex were also included as nodes in the analysis, as covariates. Thus, the thickness of the edge represents the strength of the unique shared association between each couple of nodes, after adjusting or all other symptoms in the network, as well as gender and age, and is hypothesized to estimate the strength of the causal relationship between each couple of symptoms. As conventional, we selected the significant relationships between each couple of variables based on 95% Credible Intervals (95%CrI) not crossing the zero, which is analogue to selecting associations based on a statistical significance level of *p* value lower than 0.05. We report weighted edges to indicate the strength of the connection, estimated by a partial correlation coefficient: Analyses were conducted with the 2.0.3 BGGM package for R (Williams, 2021).

## 4 Results

Network analyses revealed significant connections between nodes related to senses (AASP nodes), the task (comfort), and psychopathological nodes representing the SAFA protocol subscales. The task related to comfort showed correlations with specific scales, indicating associations with sensory experiences and psychopathological factors.

**Figure 1:**
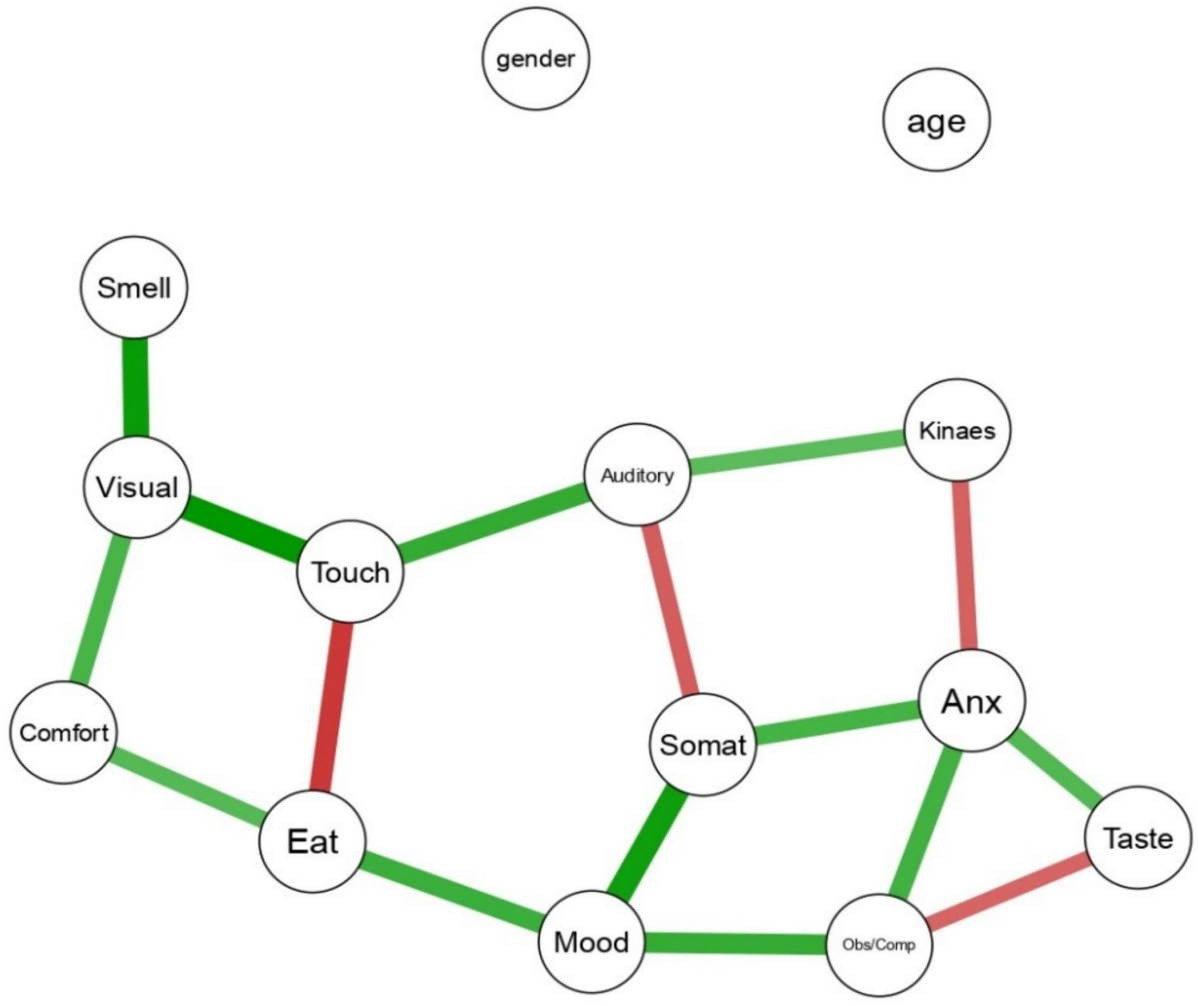
Network analysis.

In particular, the task related to comfort demonstrated a correlation with the visual scale of the Adolescent/Adult Sensory Profile (AASP; edge strength = 0.301). Additionally, a correlation was observed with the Eating Disorder scale of the SAFA protocol (edge strength = 0.277). These findings suggest that individuals’ comfort perceptions during the comfort distance estimation task are linked to both visual sensory experiences and aspects related to eating disorder symptom as assessed by the SAFA protocol. Touch also negatively correlated with Eating disorder symptoms (edge strength = −0.328). Touch is one of the major sensory modalities that contribute to the construction and maintenance of body representations. In eating disorders, tactile perception may be altered, leading to distortions in body perception (Risso and Bassolino, 2022), a worsening of tactile perception might be associated with an increase in body image distortion. No connection was found between age or gender and any other node (Table 2; Figure 1).

**Table 2:** Weighted adjacency matrix (edge strengths).

	Comfort	Taste	Kinaes	Visual	Auditory	Smell	Touch	Anx	Mood	Obs/Com	Eat	Somat
Comfort				0.301							0.277	
Taste								0.282		−0.251		
Kinaes					0.267			−0.258				
Visual	0.301					0.403	0.421					
Auditory			0.267				0.333					−0.265
Smell				0.403								
Touch				0.421	0.333						−0.328	
Anx		0.282	−0.258							0.312		0.307
Mood										0.333	0.320	0.395
Obs/Com		−0.251						0.312	0.333			
Eat	0.277						−0.328		0.320			
Somat					−0.265			0.307	0.395			

## 5 Discussion

We explored the relationship between peripersonal space, sensory profiles and psychiatric symptoms in healthy adolescents. We found a positive association between the extent of PPS and subclinical symptoms related to eating disorders. Specifically, the greater the extent of peripersonal space, the more participants reported difficulties with eating behavior. Contrary to our hypotheses, we did not find a connection with symptoms of anxiety possibly due to… To our knowledge, this is the first time that the association between peripersonal space and psychopathology is assessed in adolescent, exploring the link with sensory processes.

PPS is an essential component of bodily self-consciousness and its evaluation allows us to navigate through space, perform actions and defensive purposes such as protecting the body while moving (Brozzoli et al., 2012). In our study we assessed PPS as the interpersonal distance between human-like avatars in a digitized task, in particular the distance to which the participant felt the avatars would feel comfortable. Interpersonal Space is a fundamental component of PPS and has a key role in social interactions (Coello and Cartaud, 2021). In line with these results, previous studies have discussed an involvement of peripersonal space evaluation in psychopathology such as phobias and trauma-related disorders (Rabellino et al., 2020). As in our study, Nandrino and colleagues (Nandrino et al., 2017) have also shown a relation between PPS and eating disorder symptomatology in adults. Our results demonstrate that a similar relation is already present in adolescents and therefore, suggests that socio-emotional, as reflect by difficulties regulating an appropriate interpersonal distance may be possible marker of the development of an eating disorder symptoms.

Interestingly, the observed impact on PPS regulation appears to be intricately tied to the social significance of the stimuli. This is underscored by the work of Nandrino and colleagues, who found that alterations in PPS were not evident when the stimulus was an inanimate object rather than a person (Nandrino et al., 2017). In our study, where adolescents from a non-clinical population were involved, the results suggest that changes in PPS evaluation could be indicative of a trait symptom associated with eating disorder symptoms. Possibly, adolescents prone to develop an eating disorder seem sensitive to the interpersonal space and its emotional as well motivational relevance; its sensitivity may serve a self-protective mechanism. The inclination towards larger interpersonal distances might reflect underlying social insecurity, indicative of attachment difficulties often observed in eating disorders (Tasca et al., 2009). Eating disorders, are complex mental health conditions influenced by various factors, including sensory processing and the interaction between different sensory modalities. Sensory experiences related to taste, smell, texture, and interoception (internal body sensations) can contribute to the development and maintenance of these disorders. Visual processing plays a crucial role in body image perception. Individuals with eating disorders often have a distorted body image, perceiving themselves as overweight even when underweight. This visual distortion can drive restrictive eating behaviors and body-checking rituals. At the same time, auditory and visual exposure to media promoting thin ideals and diet culture can reinforce negative body image and eating disorder behaviors. Constant exposure to unrealistic body standards can perpetuate feelings of inadequacy and the desire to achieve an unattainable body shape. In our study, we observed a positive correlation between the task related to comfort and the visual scale of the Adolescent/Adult Sensory Profile (AASP), as well as with the Eating Disorder scale of the SAFA protocol. These findings suggest that sensory processing patterns, particularly in the visual modality, are linked to how comfortable individuals feel in specific tasks. Moreover, the correlation with the SAFA Eating Disorder scale indicates that sensory processing may play a role in the manifestation and maintenance of eating disorder symptoms. These results highlight the importance of assessing sensory processing in individuals at risk of developing eating disorders. By understanding their sensory profiles, clinicians can develop more targeted and effective interventions. For example, individuals who are highly sensitive to visual stimuli may benefit from therapeutic environments that minimize visual distractions and stressors. Overall, our findings underscore the complex interplay between sensory processing and psychopathology. However, it’s crucial to note that our study represents a snapshot, and to truly comprehend the dynamics at play, longitudinal studies are imperative. Such studies would help unravel the complex relationship between sensory processing, PPS evaluation, and their connections to socio-emotional deficits across the diverse spectrum of eating disorder symptomatology. Nonetheless, our research underscores the critical importance of fortifying the interpersonal and emotional capacities of adolescents. The significance lies in cultivating their ability to navigate social interactions and emotional challenges effectively. Negative social evaluation in adolescents prone to develop an eating disorder, plays a pivotal role, via its detrimental impact on self-evaluation and associated affect, in the development and maintenance of these disorders. Our results, might empirically support the use of interpersonal psychotherapy (IPT), in giving primacy to negative social evaluation in the maintenance of eating disorder symptoms in adolescents at risk (Rieger et al., 2010). Moreover, strategies such as exposure therapy and the integration of virtual reality emerge as promising interventions to alleviate social anxiety and enhance self-assurance in various social settings. Addressing these facets in the early stages of adolescence holds the potential to contribute significantly to the prevention and improved management of eating disorder symptoms within this susceptible population.

## 6 Limits of the study

This study is strengthened by a non-invasive assessment of PPS and by a multivariate approach, that allows to investigate in a single analysis the associations between several aspects pertaining psychopathology, PPS and sensory processing. However, the study presents limitations. First, the sample size is relatively small. Such a number of participants is more than the average in the literature on peripersonal space, but may be underpowered to detect smaller associations, especially those that might be encountered among non-clinical populations. Thus, further studies are needed to confirm these findings, and to rule out an association between PPS extent and anxiety in the adolescent population. Nonetheless, given the recruitment strategy and high adherence rates, the sample may be considered reasonably representative of the reference population of mid-schooler adolescents. Second, the study design is cross-sectional and is unable to capture changes over time, or to establish the direction of causality. Further longitudinal studies might elucidate how PPS, sensory processing and psychopathology develop over time.

Third, the assessment of interpersonal PPS was conducted with a virtual avatar task conducted within a controlled laboratory environment. While this method is non-invasive and efficient, using a simulated condition might limit the ecological validity of results, as real-world dynamics may differ from those simulated in the laboratory. Further study are necessary to examine the reliability of the Avatar PPS assessment with that of more invasive techniques.

Fourth, while we adjusted the analyses for gender, the sample was relatively imbalanced. Interpersonal dynamics present substantial gender-related differences, highlighting the need of conducting gender-specific analyses in future, larger studies.

Limited psychopathology evaluation: while the SAFA protocol covers a broad range of internalizing symptoms, the evaluation of psychopathological aspects could be further enhanced by including externalizing symptoms or other relevant measures for a comprehensive assessment.

This study adopts an exploratory approach, as we did not have *a priori* hypotheses. The exploratory nature of the study highlights the need for further research to investigate more precisely the correlation between eating disorder symptoms, their features, and PPS extension.

## 7 Conclusion

In conclusion, the extent of interpersonal space was associated with dimensions of sensory processing and with features of eating disorders in healthy adolescents. This association suggests that bodily processes may be crucially involved in the development of social, interpersonal and behavioral skills. Adolescents face known difficulties to develop a healthy bodily-self, necessary to navigate the complexities of interpersonal relationships and emotional well-being. PPS assessment and Interventions like exposure therapy might facilitate adolescents to recognize difficulties of social interactions and help them to gradually build confidence.

Similarly, virtual reality offers a controlled environment to practice and enhance social skills, building a sense of self-efficacy in real world scenarios.

## Author’s note

On behalf of MNESYS-Mood and Psychosis Sub-Project (spoke 5 o spoke 6).

## Data Availability

The raw data supporting the conclusions of this article will be made available by the authors, without undue reservation.
